# A Chronic Cough and Something More: An Unusual Presentation of Hepatocellular Carcinoma

**DOI:** 10.7759/cureus.37300

**Published:** 2023-04-08

**Authors:** Maria Cristina Cuartas-Mesa, Maria E Romero Noboa, Yasmine Choroomi, Badri Aryal, Akash Venkataramanan, Rafaella Ferreira de Araujo Litvin

**Affiliations:** 1 Internal Medicine, John H. Stroger, Jr. Hospital of Cook County, Chicago, USA; 2 Internal Medicine, Rosalind Franklin University of Medicine and Science, Chicago, USA

**Keywords:** transarterial chemoembolization (tace), liver cancer, diaphragm irritation, chronic cough, hepatocellular carcinoma (hcc)

## Abstract

Liver cancer is one of the main contributors to cancer-related death and is the fifth most frequent cancer worldwide. Hepatocellular carcinoma (HCC), the most common of liver cancers, is most frequently diagnosed incidentally during routine imaging in high-risk patients with cirrhosis. However, patients with advanced disease may present with upper abdominal pain, early satiety, weight loss, and a palpable upper abdominal mass. We describe a case of HCC in a 69-year-old male presenting exclusively with an intractable cough, which improved after transarterial chemoembolization-raising awareness of the importance of having a systematic and physiopathology-based approach to chronic cough to have an adequate diagnosis and treatment.

## Introduction

Liver cancer is one of the main contributors to cancer-related death, with hepatocellular carcinoma (HCC) being the most common type [1]. HCC generally develops in the setting of cirrhosis, which occurs worldwide primarily due to hepatitis B and C viruses and alcohol. However, up to 20% of cases may occur in patients without cirrhosis but with other predisposing conditions such as chronic hepatitis [2]. The rising prevalence of obesity and non-alcoholic hepatic steatosis also seems to be contributing to an increase in the incidence of HCC [3].

Most of the time, HCC is diagnosed incidentally during routine imaging in high-risk patients with cirrhosis. However, patients with advanced disease may present with upper abdominal pain, early satiety, weight loss, and a palpable upper abdominal mass. The standard treatment for HCC is surgical excision. Advanced, unresectable tumors can also be managed by transarterial embolization and immunotherapy [4].

We describe a rare case of HCC in a 69-year-old male presenting with an intractable cough that improved after transarterial chemoembolization. We also review the common presentations of HCC and the physiologic mechanisms of cough. We aim to assist clinicians in broadening their differential diagnosis for patients presenting with chronic cough, as well as lowering the threshold of suspicion for HCC in patients who might be at risk but present with unusual symptoms.

## Case presentation

A 69-year-old male with a history of hypertension and a remote history of smoking presented to our institution with a subacute cough and respiratory distress. The patient had experienced three weeks of dry cough, intermittent subjective fevers, and worsening shortness of breath, prompting his ED visit. The patient was hypoxic, tachypneic, and had stridor. He received non-invasive ventilation, nebulized bronchodilator therapy, steroids (intravenous and oral), intravenous magnesium, and antibiotic therapy for possible upper respiratory infection. Computed tomography (CT) pulmonary angiogram excluded a pulmonary embolism and showed no parenchymal changes. Cardiac echocardiogram, brain natriuretic peptide (BNP), and troponin were all within normal limits. A respiratory viral panel, coronavirus disease (COVID) polymerase chain reaction (PCR), and blood and sputum cultures were all negative. Laryngoscopy evaluation performed by otolaryngology was also unremarkable. His respiratory status and stridor improved within a few days, but the cough persisted. Etiology at this point was unclear, and laryngospasm related to gastroesophageal reflux disease or exacerbation of previously undiagnosed chronic obstructive pulmonary disease (COPD) was considered. He was discharged on prednisone, pantoprazole, and an inhaled corticosteroid with a long-acting beta agonist.

He returned to the hospital one month later with a worsening dry cough, now present for a total of eight weeks. He reported severe paroxysms of cough limiting his daily activities, which had become associated with non-pleuritic, non-positional chest pain. He had no associated dyspnea, dyspepsia, or reflux symptoms. Vital signs were normal, and the lung exam was unremarkable. He was admitted and again treated for chronic obstructive pulmonary disease (COPD) exacerbation with no improvement in the cough and later started on codeine and gabapentin as an attempt at symptom control.

**Figure 1:**
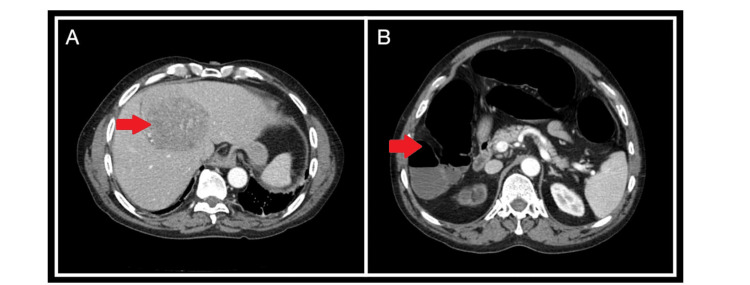
CT abdomen CT of the abdomen showing the liver lesion (A) and dilated loops of the bowel (B).

The patient developed constipation, abdominal pain, and distension on the fifth day of admission. Along with signs of ileus, a CT abdomen (Figure 1) and pelvis incidentally showed an 8.6 x 11.6 cm hypervascular mass on segment 4A/8 of the liver, which abuts the diaphragm. Alpha-fetoprotein (AFP) levels returned elevated at 303.6 ng/mL. The hepatitis C viral load was 388,60 2IU/L (newly diagnosed), and a triple-phase liver CT confirmed hepatocellular carcinoma features (LI-RADS 5), without portal invasion.

The patient was classified as BCLA stage B (intermediate). Given the size and location of the tumor, he was not considered a surgical candidate. The cough was partially controlled with gabapentin, the ileus resolved after bowel rest, and the patient was referred for transarterial chemoembolization (TACE) for the HCC. On follow-up two weeks after undergoing TACE, his cough had entirely resolved.

## Discussion

Liver cancer is the fifth most common cancer and the fourth leading cause of cancer-related deaths worldwide [5], with HCC accounting for more than 80% of primary liver cancer cases [3], making it imperative to focus on prevention and early diagnosis. Clinical presentation depends on the underlying etiology and the stage of the disease and can range from asymptomatic to overt signs of decompensated cirrhosis, abdominal pain, a palpable mass, weight loss, or early satiety [6]. Treatment of HCC is based on staging. There are different staging systems, among which the Barcelona Clinic Liver Cancer (BCLC) system is widely accepted; this takes into account the performance status, the tumor characteristics (size, number of tumors, vascular involvement, presence of metastases), and the Child-Pugh score. Early-stage cancer is treated with radiofrequency ablation (RFA), liver resection, and liver transplantation. In contrast, intermediate-stage HCC is treated with palliative treatments such as transarterial chemoembolization (TACE) or transarterial radioembolization (TARE) [4]. Advanced-stage HCC is treated with multikinase inhibitors such as sorafenib.

Cough is a reflex mechanism of the airway tract that helps clear the upper airways. It is typically triggered by irritation of cough receptors, which are sensory vagal fibers found mainly in the airway but also in the external auditory canals, eardrums, paranasal sinuses, pharynx, pleura, pericardium, and diaphragm [7]. A cough lasting for more than eight weeks is considered persistent or chronic. Multiple etiologies can cause chronic cough, with some of the most common being asthma, gastro-esophageal reflux disease, eosinophilic bronchitis, postnasal drip syndrome, chronic obstructive pulmonary disease, pulmonary fibrosis, and bronchiectasis [8]. As our patient was a former smoker, his respiratory symptoms were initially managed as a possible obstructive airway process. However, his cough persisted despite inhaled therapies and corticosteroids, he had no chronic respiratory symptoms prior to the cough, and lung imaging showed no signs of COPD. The fact that the cough completely resolved after he underwent chemoembolization suggests a possible causal relationship between the liver mass and the chronic cough.

This would be an extremely rare presentation, and there is only one prior report of HCC presenting with a cough [9]. Other cases of chronic cough thought to be secondary to diaphragm irritation from liver diseases have been described in the literature, including a case of a giant cavernous hemangioma, where the cough resolved after hepatic lobectomy [10], and one of a pyogenic liver abscess, where the cough resolved after percutaneous drainage [11].

## Conclusions

These cases further support the proposed cough pathophysiology but do not prove causality. However, after ruling out common conditions of chronic cough through diagnostic testing for empiric treatment, it is worthwhile considering rare causes, such as HCC, and even consider further imaging and testing in a relevant clinical context. A systematic approach to evaluating chronic cough, considering the underlying physiologic mechanisms, is fundamental to accurately determining the potential cause and targeting the treatment.
